# Transcriptomics *de novo* sequencing data of *Messastrum gracile* SE-MC4 under exponential and stationary growth stages

**DOI:** 10.1016/j.dib.2021.107607

**Published:** 2021-11-19

**Authors:** C. L. Wan Afifudeen, Saw Hong Loh, Li Lian Wong, Ahmad Aziz, Kazutaka Takahashi, Mohd Effendy Abd Wahid, Thye San Cha

**Affiliations:** a Faculty of Science and Marine Environment, University Malaysia Terengganu, Kuala Nerus, Terengganu 21030, Malaysia; b Satreps-Cosmos Laboratory, Central Laboratory Complex, University Malaysia Terengganu, Kuala Nerus, Terengganu 21030, Malaysia; c Institute of Marine Biotechnology, University Malaysia Terengganu, Kuala Nerus, Terengganu 21030, Malaysia; d Department of Aquatic Bioscience, Graduate School of Agricultural and Life Sciences, The University of Tokyo, 1-1-1, Yayoi, Bunkyo-ku, Tokyo, 113-8657, Japan; e Faculty of Fisheries and Food Science, Universiti Malaysia Terengganu, Kuala Nerus, Terengganu 21030, Malaysia

**Keywords:** Non-model microalga, Cell proliferation, Biodiesel, Next generation sequencing data

## Abstract

*Messastrum gracile* SE-MC4 is a non-model microalga exhibiting superior oil-accumulating abilities. However, biomass production in *M. gracile* SE-MC4 is limited due to low cell proliferation especially after prolonged cultivation under oil-inducing culture conditions. Present data consist of next generation RNA sequencing data of *M. gracile* SE-MC4 under exponential and stationary growth stages. RNA of six samples were extracted and sequenced with insert size of 100 bp paired-end strategy using BGISEQ-500 platform to produce a total of 59.64 Gb data with 314 million reads. Sequences were filtered and *de novo* assembled to form 53,307 number of gene sequences. Sequencing data were deposited in National Center for Biotechnology Information (NCBI) and can be accessed via BioProject ID PRJNA552165. This information can be used to enhance biomass production in *M. gracile* SE-MC4 and other microalgae aimed towards improving biodiesel development.

## Specifications Table

**Table:** 

Subject	Molecular Biology
Specific subject area	Transcriptome Data
Type of data	Transcriptome data of non-model microalga *Messastrum gracile* SE-MC4
How data were acquired	100 bp paired-end transcriptome sequencing of *M. gracile* SE-MC4 using BGISEQ-500 at Beijing Genome Institute, China.
Data format	Raw sequences: FASTQ Filtered and assembled: FASTQ
Parameters for data collection	RNA extracted from *in vitro* cultivated *M. gracile* SE-MC4; harvested at exponential and stationary growth phase
Description of data collection	Cell was grown under pure and homogenous cell culture (axenic environment). RNA was extracted from harvested cell for sequencing purposes. Output from sequencing were assembled using Trinity algorithm.
Data source location	Institution: 1) Satreps-Cosmos Laboratory, Central Laboratory Complex, Universiti Malaysia Terengganu, 21030 Terengganu, Malaysia 2) Institute of Marine Biotechnology, Universiti Malaysia Terengganu City/Town/Region: Kuala Nerus, Terengganu Country: Malaysia Latitude and longitude (and GPS coordinates) for collected samples/data: 5° 24′ 46.4" N 103° 05′ 10.2" E (Kuala Terengganu, Terengganu)
Data accessibility	Repository name: National Center for Biotechnology Information (NCBI) Data identification number: BioProject ID PRJNA552165 Direct URL to data: https://www.ncbi.nlm.nih.gov/bioproject/PRJNA552165
Related research article	C.L. Wan Afifudeen, A. Aziz, L.L. Wong, K. Takahashi, T. Toda, M.E. Abd Wahid, T.S. Cha, 2021. Transcriptome-wide study in the green microalga Messastrum gracile SE-MC4 identifies prominent roles of photosynthetic integral membrane protein genes during exponential growth stage. Phytochemistry. 192: 112936. DOI:https://doi.org/10.1016/j.phytochem.2021.112936

## Value of the Data

• Transcriptome sequences data of *Messastrum gracile* under different growth stages can be used for growth and developmental studies for higher biomass productivity in microalgae.
• Transcriptome experts and biodiesel scientists can use this sequencing data for data mining (targeted gene) for gene transformation purposes to enhanced biomass productivity in microalgae for biodiesel.
• This data provide an insight on transcriptome profiles during rapid developmental process thus can be used in overexpression studies for high biomass cultivation of microalgae for biodiesel.

## 1 Data Description

This report consist of complete *M. gracile* transcriptome data under cell exponential growth stage (cell proliferation) and stationary growth stage (cell growth limitation). A total of 314.82 million reads (total base 59.64 Gb) were produced from six samples with an average Q30 (Phred score) of 88.46% (Table 1). Raw sequences were filtered and produced an average of 95.03% of clean reads.

**Table 1:** Sequencing quality data of TWAS from *M. gracile* SE-MC4 using BGISEQ-500 platform.

Sample	Total Raw Reads (Million)	Total Clean Bases(Gb)	Q20 (%)	Q30 (%)	Clean Reads (%)
1D _F2_R1	52.47	4.99	96.38	87.90	95.12
1D _F2_R2	52.47	4.97	96.42	87.98	94.79
1D _F2_R3	52.47	4.96	96.47	88.27	94.50
12D_F2_R1	52.47	4.98	96.41	88.09	94.82
12D_F2_R3	52.47	4.95	96.30	87.89	94.29
12D_F2_R2	52.47	4.97	96.16	87.46	94.70
Total/Average	314.82	59.64	96.57	88.46	95.03

*Note:* Samples 1D_F2_R1/R2/R3 represent three biological replicates for exponential growth (day 1) phase cultures. Samples 12D_F2_R1/R2/R3 represent three biological replicates for stationary (day 12) growth phase cultures.

Transcriptome sequences were deposited to NCBI under BioProject ID PRJNA552165 with six different BioSample which were SAMN12670086, SAMN12670087, SAMN12670088, SAMN12670089, SAMN12670090, and SAMN12670091 accordingly (Table 2). Filtered sequences were *de novo* assembled using Trinity to form 53,307 gene transcripts with mean length of 623 bp, N50 of 928, and average of 71.53% GC content (Table 3). Distribution of gene transcripts based on length show that most transcripts were between 200 and 300 bp (9876 to 10718 gene transcripts) in all six samples (Table 4). Furthermore, exponential growth samples produce between 37171 and 38254 gene transcripts while stationary growth samples produce between 34344 to 35344 gene transcripts. Details on experimental design and sequence are described in experimental design, materials and methods section.

**Table 2:** Sequence accession numbers (BioProject, BioSample) and directory links.

Samples	Accession number	Links
*M. gracile*SE-MC4	PRJNA552165(BioProject ID)	https://www.ncbi.nlm.nih.gov/bioproject/PRJNA552165
1D_F2_R1	SAMN12670086	https://www.ncbi.nlm.nih.gov/biosample/ SAMN12670086
1D _F2_R2	SAMN12670087	https://www.ncbi.nlm.nih.gov/biosample/ SAMN12670087
1D _F2_R3	SAMN12670088	https://www.ncbi.nlm.nih.gov/biosample/ SAMN12670088
12D_F2_R1	SAMN12670089	https://www.ncbi.nlm.nih.gov/biosample/ SAMN12670089
12D_F2_R2	SAMN12670090	https://www.ncbi.nlm.nih.gov/biosample/ SAMN12670090
12D_F2_R3	SAMN12670091	https://www.ncbi.nlm.nih.gov/biosample/ SAMN12670091

*Note:* Samples 1D_F2_R1/R2/R3 represent three biological replicates for early exponential growth (day 1) phase cultures. Samples 12D_F2_R1/R2/R3 represent three biological replicates for early stationary (day 12) growth phase cultures.

**Table 3:** Sequencing quality data of WTS from *M. gracile* SE-MC4.

Sample	Total Number	Total Length	Mean Length	N50	N70	N90	GC (%)
1D _F2_R1	50,032	31,620,070	631	940	537	268	71.42
1D _F2_R2	49,281	31,029,976	629	934	541	268	71.28
1D _F2_R3	51,229	32,659,375	637	955	550	269	71.64
12D_F2_R1	46,263	29,294,862	633	952	549	266	71.58
12D_F2_R2	46,429	29,723,323	640	971	554	269	71.58
12D_F2_R3	47,847	29,451,981	615	921	524	258	71.70
Total/Average	53,307	-	623	928	537	265	71.53

*Note:* Samples 1D_F2_R1/R2/R3 represent three biological replicates for early exponential growth (day 1) phase cultures. Samples 12D_F2_R1/R2/R3 represent three biological replicates for early stationary (day 12) growth phase cultures.

**Table 4:** Sequencing quality data of WTS from *M. gracile* SE-MC4.

Unigenessize bp	1D_F2_R1	1D_F2_R2	1D_F2_R3	12D_F2_R1	12D_F2_R2	12D_F2_R3
300	10625	10534	10718	9918	9876	10670
400	6082	5908	6023	5237	5277	5538
500	3880	3769	3801	3365	3376	3310
600	2729	2651	2725	2394	2401	2511
700	2105	2091	2162	1932	1877	1908
800	1723	1757	1777	1615	1576	1622
900	1425	1423	1422	1292	1302	1303
1000	1182	1229	1318	1105	1106	1113
1100	1038	1093	1090	1046	991	1031
1200	928	906	912	847	860	839
1300	774	785	813	710	774	748
1400	724	709	718	656	675	627
1500	575	576	637	572	552	546
1600	493	453	546	473	481	437
1700	477	426	455	424	405	414
1800	403	421	425	402	394	380
1900	310	319	358	328	310	298
2000	305	298	342	281	304	280
2100	282	260	268	240	237	242
2200	223	230	223	211	285	207
2300	197	183	197	185	185	196
2400	171	142	194	143	157	175
2500	139	150	155	150	146	145
2600	126	126	136	109	119	111
2700	109	109	110	90	102	97
2800	117	71	111	98	94	88
2900	85	74	79	76	91	82
3000	69	57	74	54	71	66
>=3000	438	421	465	391	410	360
**Total**	37734	37171	38254	34344	34434	35344

## 2 Experimental Design, Materials and Methods

### 2.1 Sample preparation

*M. gracile* SE-MC4 cell was retrieved from microalgae stock culture collection at Universiti Malaysia Terengganu [1]. Fresh *M. gracile* SE-MC4 inoculum was initiated from a single colony solid medium and transferred into axenic F2 liquid medium. Fresh cells were then introduced to nitrate starvation (treatment) and nitrate sufficient (control) culture medium. Cells were grown until reach stationary growth stage. Cells from exponential (Day 1) and stationary (Day 12) were harvested using centrifuge for TWAS [2]. RNA was extracted from cells using GF-1 Total RNA Extraction Kit (Vivantis, Malaysia) and all procedures were followed as mention in manufacturer guide manual [3,4].

### 2.2 RNA sequencing and *de novo* assembly

Library preparation and sequencing were conducted as mention in Wan Afifudeen et al., [5]. Library preparation was built based on BGISEQ-500 PE100 strategy. Firstly, mRNA was enriched using Oligo dT selection and rRNA removal via depletion process. Then, RNA was fragmented into small length before cDNA formation via reverse transcript process. After that, adaptors were ligated into the cDNA and further amplified before denatured and cyclized into DNA Nanoballs (DNBs). DNBs were then sequenced using BGISEQ-500 platform (Beijing Genome Institute, China) [6]. Raw sequence was trimmed and filtered before assembled using Trinity v2.06 to form contigs or gene transcripts [7]. Phred value of Q20 and reads longer than 200 bp were used as baseline for reads selection for assembly.

### 2.3 Sequence deposition

RNA sequence data were deposited to NCBI under submission portal platform via https://www.ncbi.nlm.nih.gov/submission/. Submission of RNA sequence data was made under BioProject ID PRJNA552165 (Table 3).

## Ethics Statement

Work does not involved any human subjects, animal experiments or collection of data via social media platform.

## CRediT authorship contribution statement

**C. L. Wan Afifudeen:** Conceptualization, Methodology, Software, Data curation, Writing – review & editing. **Saw Hong Loh:** Conceptualization. **Li Lian Wong:** Conceptualization. **Ahmad Aziz:** Conceptualization. **Kazutaka Takahashi:** Conceptualization. **Mohd Effendy Abd Wahid:** Conceptualization. **Thye San Cha:** Conceptualization, Writing – review & editing.

## Declaration of Competing Interest

The authors declare that they have no known competing financial interests or personal relationship that could have appeared to influence the work reported in this paper.
